# Simple Diagnostic Tests for Subungual Pigmentation

**DOI:** 10.1155/2009/278040

**Published:** 2010-02-17

**Authors:** Shardul Poudyal, David J. Elpern

**Affiliations:** ^1^Department of Family Medicine, University of Colorado, 3936 Main Street no. 202 Westminster, Denver, CO 80031, USA; ^2^12 Meadow Street, Williamstown, MA 01267, USA

## Abstract

Subungual pigmentation can have benign and malignant etiologies. A common and important differential diagnosis is between subungual hematoma and subungual acrolentiginous melanoma. We have introduced Dr. Eckert Haneke's technique and our Hydrogen Peroxide modification for distinguishing these entities clinically. Dr. Haneke's technique uses the hemocult reaction to detect hematoma from the specimen, while our modification uses Hydrogen Peroxide to clear the hematoma and make the decision clinically. Both are minimally invasive techniques which can be performed without pain. Often these procedures spare the patient an unnecessary tissue biopsy with its morbidity and discomfort. Importantly, they reassure the patient that he or she has a benign disorder.

## 1. Introduction

Subungual pigmentation can have benign and malignant etiologies. A common and important differential diagnosis is between subungual hematoma and subungual melanoma [1]. The distinction can be difficult even with a dermoscope. A number of years ago, Dr. Eckart Haneke pioneered a procedure to differentiate these entities clinically. We would like to describe the Haneke procedure [2–4] and a recent modification we developed. 

**Figure 1:**
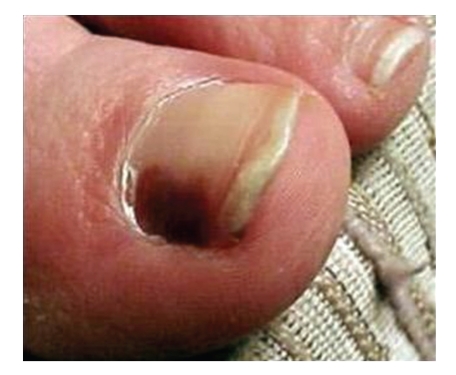
Subungual pigmentation.

A patient presents with a two-month history of a brownish pigmentation of the great toe nail (Figure 1). While some recommend biopsy of the nail bed to determine if this is neoplastic or traumatic, a simpler technique exists.

## 2. Haneke Procedure [2–4]

**Figure 2:**
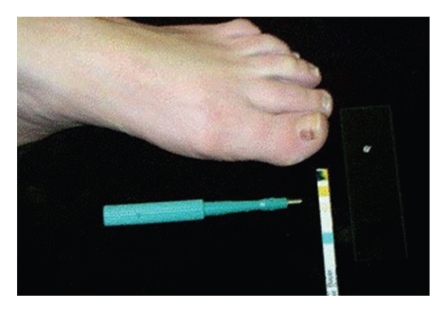
Trocar, Glass slide with scrapped nails, and Hemocult stick showing the result.

1. Soften the nail by soaking the foot in lukewarm water for 20 to 30 minutes.
2. Carefully, drive a 3 mm punch through the softened nail. Local anesthesia is not used because we want the patient's pain sensation to warn us whether we are getting close to the nail bed. Be cautious not to traumatize the nail bed.
3. Remove the cut edge of the nail with a small curved scissors and observe the underside of the nail biopsy and nail bed.
4. Take the trepanned section of nail and scrape the bottom.
5. Place the scrapings on a glass slide and add a drop of water and mix.
6. Then rub a Hemocult stick (or urinalysis reagent strip) in the mixture. If the strip changes color, then this indicates hematoma. If it does not, then it is negative for blood and a melanocytic lesion is likely (see Figure 2).
7. If one does not find blood after scraping the underside of the trepanned section of the nail, but some pigmentation is still visible, one can add a tiny drop of water into the drilled hole. Then after waiting for 5 to 10 minutes, a Hemocult strip can be applied directly.

## 3. Modified Haneke Procedure

**Figure 3:**
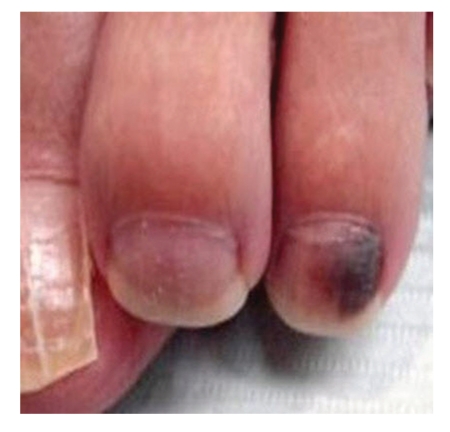
Subungual pigmentation.

**Figure 4:**
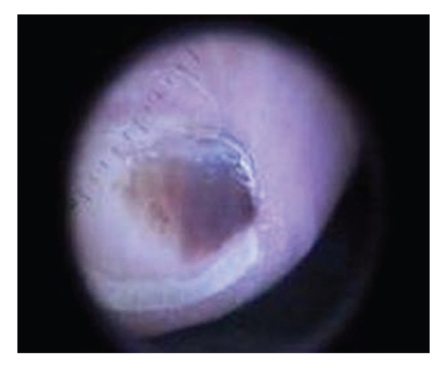
Dermoscopy before 3 mm biopsy.

**Figure 5:**
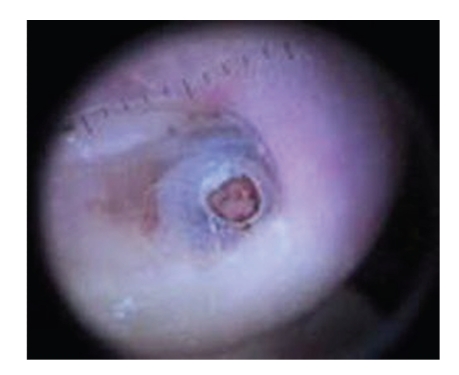
Dermoscopy after 3 mm Punch biopsy and H_2_O_2_ defect.

**Figure 6:**
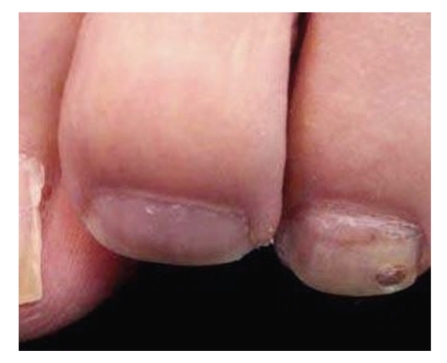
Followup picture of Figure 3 after four weeks. Notice the complete resolution of subungual hematoma leading to clearing of nail bed.

**Figure 7:**
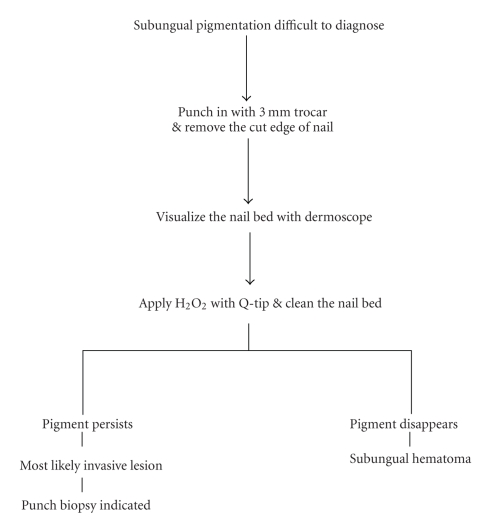
Flow chart of modified Haneke procedure.

1. Soften the nail by soaking the foot in lukewarm water for 20 to 30 minutes.
2. Carefully, drive a 3 mm punch through the pre-soaked nail. Local anesthesia is not used because we want the patient's pain sensation to warn us if whether are getting close to the nail bed. Be cautious not to traumatize the nail bed.
3. Remove the cut edge of the nail and observe the underside of the nail biopsy and nail bed. It is advantageous to use dermoscope while evaluating the nail bed as unassisted eyes can sometimes miss the diagnosis.
4. Apply a drop or two of hydrogen peroxide (H_2_O_2_) with a Q-tip to clean the nail bed. If the pigmentation is due to old blood (subungual hematoma), then a normal appearing nail bed will be seen after cleaning with hydrogen peroxide. If, after cleaning with hydrogen peroxide, pigment persists on the nail bed, then it is likely due to melanocytes and a nail bed biopsy is indicated (see Figures 3–7).

## 4. Precaution and Special Attention

1. Do not cut into the nail bed as it can cause bleeding which not only can be painful to the patient but also gives a false positive reaction.
2. Do not overuse hydrogen peroxide so as to damage the nail bed or its surrounding area.
3. Some subungual tumors including melanomas can bleed secondary to nail bed invasion. Thus, a hasty conclusion should not be drawn and clinical judgment should be exercised.
4. A followup visit two to three weeks after the procedure will help to confirm the initial findings (Figure 6).

## 5. Conclusion

Both the Haneke technique and the Hydrogen Peroxide modification can be used to effectively differentiate an old subungual hematoma from a subungual melanoma. These minimally invasive techniques are inexpensive, do not require special training, and can be performed without specialized equipment in almost any clinical setting. Often they will spare the patient an unnecessary nail bed biopsy with its morbidity and discomfort. Importantly, if positive for blood, they reassure the patient that he or she has a benign disorder.
